# PIPE: a protein-protein interaction prediction engine based on the re-occurring short polypeptide sequences between known interacting protein pairs

**DOI:** 10.1186/1471-2105-7-365

**Published:** 2006-07-27

**Authors:** Sylvain Pitre, Frank Dehne, Albert Chan, Jim Cheetham, Alex Duong, Andrew Emili, Marinella Gebbia, Jack Greenblatt, Mathew Jessulat, Nevan Krogan, Xuemei Luo, Ashkan Golshani

**Affiliations:** 1 School of Computer Science, Carleton University, Ottawa, Canada; 2 Department of Mathematics and Computer Science, Fayetteville State University, Fayetteville, USA; 3 Department of Biology, Carleton University, Ottawa, Canada; 4 Banting and Best Institute of Medical Research, Department of Medical Genetics and Microbiology, University of Toronto, Toronto, Canada

## Abstract

**Background:**

Identification of protein interaction networks has received considerable attention in the post-genomic era. The currently available biochemical approaches used to detect protein-protein interactions are all time and labour intensive. Consequently there is a growing need for the development of computational tools that are capable of effectively identifying such interactions.

**Results:**

Here we explain the development and implementation of a novel Protein-Protein Interaction Prediction Engine termed PIPE. This tool is capable of predicting protein-protein interactions for any target pair of the yeast Saccharomyces cerevisiae proteins from their primary structure and without the need for any additional information or predictions about the proteins. PIPE showed a sensitivity of 61% for detecting any yeast protein interaction with 89% specificity and an overall accuracy of 75%. This rate of success is comparable to those associated with the most commonly used biochemical techniques. Using PIPE, we identified a novel interaction between YGL227W (vid30) and YMR135C (gid8) yeast proteins. This lead us to the identification of a novel yeast complex that here we term vid30 complex (vid30c). The observed interaction was confirmed by tandem affinity purification (TAP tag), verifying the ability of PIPE to predict novel protein-protein interactions. We then used PIPE analysis to investigate the internal architecture of vid30c. It appeared from PIPE analysis that vid30c may consist of a core and a secondary component. Generation of yeast gene deletion strains combined with TAP tagging analysis indicated that the deletion of a member of the core component interfered with the formation of vid30c, however, deletion of a member of the secondary component had little effect (if any) on the formation of vid30c. Also, PIPE can be used to analyse yeast proteins for which TAP tagging fails, thereby allowing us to predict protein interactions that are not included in genome-wide yeast TAP tagging projects.

**Conclusion:**

PIPE analysis can predict yeast protein-protein interactions. Also, PIPE analysis can be used to study the internal architecture of yeast protein complexes. The data also suggests that a finite set of short polypeptide signals seem to be responsible for the majority of the yeast protein-protein interactions.

## Background

Proteins carry out the majority of the biological processes in cells. Most often, proteins accomplish this task in association with protein partners, forming stable or transient protein complexes. It is therefore generally accepted that protein-protein interactions are responsible for the cell's behaviour and its responses to various stimuli [1-3]. Further, the completion of higher eukaryotic genome projects have led to the understanding that the biological complexity underlying higher organisms is not accomplished by increasing the number of genes [4-6]. It is now thought that this complexity stems from an elevated pattern of protein-protein interactions in higher organisms [7,8]. As a consequence, charting protein-protein interaction maps remains a major goal in biological research.

A large part of post-genomic research has focused on the analysis of protein-protein interactions. Measurement, prediction and analysis of interactions between proteins have been extensively used to identify proteins that are functionally related. As a consequence, analysis of protein interaction networks has become a powerful tool to assign putative functions to previously ill-characterized proteins [2,3]. In this context, the yeast *Saccharomyces cerevisiae *has emerged as the model organism for studying functional proteomics. Very recently, we used protein interaction analysis to assign putative functions to different yeast proteins [9,10].

Protein-protein interactions can be most readily identified by protein affinity chromatography or pull-down experiments, yeast two-hybrid screens, or purifying protein complexes that have been tagged *in vivo*. These methods are all labour and time consuming and have a high cost associated with them. Each of them has inherent advantages and disadvantages. The yeast two-hybrid system has the advantage of identifying the direct interaction between protein pairs [11,12]. However, the data gathered from this method has a high (as much as 50%) rate of false positives and in the absence of other lines of evidence, this data alone may not be considered as biologically significant [13,14]. Affinity purification methods such as the *in vivo *double-tagging of protein complexes followed by purification steps using affinity chromatography, also known as tandem affinity purification (TAP tag), has the advantage of identifying complexes that really exist *in vivo *(as long as the tagged protein is not overproduced) [15,16]. However, all affinity purification methods suffer from limitations [13,14,17]. First, the addition of a tag, large or small, to the protein may change its properties, causing changes in complex stability or composition. Second, all purification methods suffer from the co-purification of "contaminating" proteins. It is often difficult to conclude whether these "contaminants" represent true endogenous partners or artificial associations induced by cell disruption. Third, during affinity purifications proteins are isolated as complexes and therefore the direct interactions between protein pairs are not readily distinguished from the indirect (via intermediates) ones.

The high cost, as well as the technical limitations associated with such biochemical approaches has resulted in a growing need for the development of computational tools that are capable of identifying protein-protein interactions. As a result, there have been a number of such tools developed over the past few years. Some of these tools are based on previously identified domains [18-20], some use similarities and sequence conservation between interacting proteins [21,22], others use the structural information of proteins [23-25] etc. The primary structure of the proteins has also been used to detect protein-protein interactions. Using a vector based learning machine it has been shown that the primary sequence of amino acids alone may successfully be used to detect protein-protein interactions [26,27]. A disadvantage of the protein-protein interaction detection tools is that they often have limited abilities to detect novel interactions and to differentiate them from false positives. A high rate of false negatives is another disadvantage associated with some of these tools.

Here, we ask the question: can novel protein-protein interactions be successfully predicted from amino acid sequences (the primary structures) alone and without any further information/prediction about the proteins? Our hypothesis is that some of the interactions between proteins are mediated by a finite number of short polypeptide sequences. These sequences may be typically shorter than the classical domains and are used repeatedly in different proteins and in different contexts within the cell. Once the interaction database is large enough to sample these sequences, it should be possible to accurately predict such protein-protein interactions. In this paper, we report on the development and implementation of a computational tool termed Protein-Protein Interaction Prediction Engine (PIPE). This engine uses the primary structure of proteins together with the available protein interaction data to predict the potential interaction between any target pairs of *S. cerevisiae *proteins.

## Results and discussion

**Figure 1:**
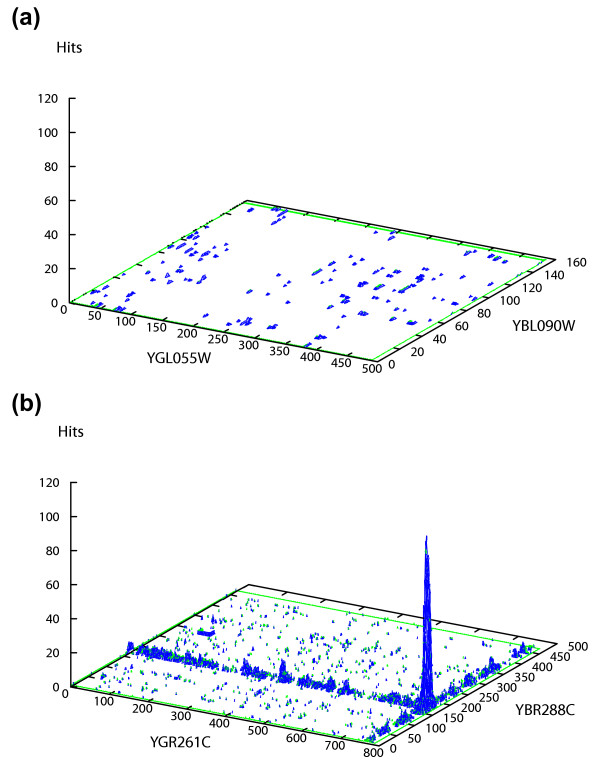
**Interaction graphs**. Two interaction graphs showing potential interaction sites for a pair of non-interacting proteins (a) and a pair of interacting proteins (b). In (a), the number of corresponding short amino acid sequences between YBL090W and YGL055W, which also co-occur in the dataset of the interacting proteins, is calculated to be very low and hence no obvious picks are detected in this graph. In (b), a sharp pick with a score of 115 indicates that the two corresponding short amino acid sequences one in middle section of YBR288C (around amino acid 140) and the other at the C-terminal end of YGR261C co-occur 115 times in the dataset of the interacting protein pairs. It is therefore hypothesized that the two proteins YBR288C and YGR261C can potentially interact.

Our protein-protein interaction prediction algorithm (PIPE) is described in detail in the Materials and Methods section of this paper. It relies on previously determined interactions for *S. cerevisiae*. For two target proteins A and B, PIPE determines the likelihood for A and B to interact. Typical PIPE output for non-interacting and interacting pairs of proteins are shown in Figure 1(a) and 1(b) respectively. A peak with a score higher than 10 indicates that PIPE is predicting an interaction.

### Ability of PIPE to detect interacting proteins

PIPE accuracy was determined by analyzing sets of known interacting pairs and expected non-interacting pairs. PIPE successfully detected 61% of interacting proteins in a randomly selected set of 100 protein pairs from the yeast protein interaction literature for which at least three different lines of experimental evidence supported the interaction (positive validation set; see Table 1). This positive validation set was selected independently of the dataset of the interacting protein pairs used by PIPE to predict interactions. This observation suggests a sensitivity of 61% and a false negative rate of 39% for PIPE data. As discussed in Materials and Methods below, the PIPE method is computationally intensive and our evaluation of PIPE took close to 1000 hours of computation time. PIPE's success rate is comparable to those obtained by *in vivo *experiments. TAP tag data are estimated to have a false negative rate of 15–50% [13] with an internal reproducibility of 70% [14], which applies only to those proteins that can be successfully tagged *in vivo *(89%) [14]. A conservative estimation of false negative rate in yeast two-hybrid screens suggests a range from 43 to 71% [13]. This finding indicates that protein interactions mediated by short polypeptide sequences may comprise the majority of protein interactions experimentally observed.

In order to evaluate the specificity and the rate of false positives associated with PIPE, a negative validation set of 100 protein pairs were gathered from the literature (see Table 2). These protein pairs are expected to not interact based on protein localization data, co-expression profiling, known direct or indirect functional or genetic relationships and the information gathered from the complete set of protein interaction datasets. 11 of these non-interacting protein pairs were predicted by PIPE to be interacting, indicating a specificity of 89% and a false/novel positive rate of 11%. It also suggests that PIPE has an overall accuracy of 75%. The low false positive rate associated with PIPE is substantially better than most experimental protein interaction detection methods. It is thought that the false/novel positive rate might be as high as 77% and 64% in TAP tag and yeast two hybrid experiments, respectively [13].

In addition to the negative validation set of 100 protein pairs discussed above, we also presented 10 pairs of random amino acid sequences of length 500 to PIPE, and PIPE detected no interactions among those 10 pairs, another indication of a low false/novel positive rate for PIPE (data not shown).

All together these data indicate that PIPE can effectively identify protein-protein interactions based on the primary structure (amino acid sequences) of proteins alone and without any previous knowledge about the higher structure, domain composition, evolutionary conservation or the function of the target proteins. This is a significant improvement over some commonly used protein-protein interaction prediction algorithms. For example, our analysis using Interpret, one of the most commonly used protein-protein interaction prediction tools [24], failed to detect the previously identified interactions for protein pairs YKL028W-YDR311W, YKR048C-YCL024W [17,28] and YOR358W-YGL237C [12] for which limited structural information is available. PIPE analysis, however, detected an interaction for these pairs with scores of 250, 160 and 100, respectively.

We note, however, that although PIPE appears to have a good specificity, it would be weak for detecting novel interactions among genome wide large-scale data sets. For example, assume that we were able to run PIPE on all (approx. 20,000,000) pairs of yeast proteins, despite PIPE's current running time. If we assume that there are approximately 50,000 true interactions, then PIPE would be expected to report approximately 30,000 true positives, 2,200,000 false positives, 17,750,000 true negatives and 20,000 false negatives. The large number of false positives compared to the number of true positives makes PIPE a weak tool for analyzing such data sets.

During the preparation of this manuscript an algorithm termed Linear Motif Discovery (LMD), which contains some parallel features to PIPE, was published elsewhere [29]. In that report the primary sequences of proteins in the database of interacting protein pairs were analyzed to identify novel protein interaction motifs. In this manner the authors identified dozens of novel interacting motif candidates. A significant difference between PIPE and this approach is that PIPE is optimized to predict the likelihood of an interaction between a given pair of proteins, whereas LMD is optimized to identify protein-protein binding motifs. The existence of a protein-protein binding motif in a pair of proteins does not indicate how likely this is going to result in an actual protein-protein interaction.

### Ability of PIPE to detect the sites of interactions between protein pairs

**Figure 2:**
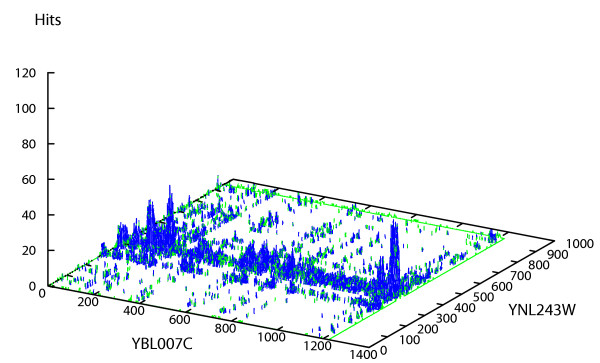
**Potential interaction sites between YNL243W and YBL007C**. PIPE can successfully determine the previously known sites of interaction between the two proteins YNL243W and YBL007C. It was previously shown that the region 310–768 in protein YNL243W is responsible for its interaction with amino acids 118–361 in protein YBL007C. Visualized by its highest pick with a score of 45, PIPE has successfully detected an interaction between YNL243W and YBL007C via their corresponding amino acid regions 350–410 and 100–250, respectively. A second highest pick with a score of 42 also suggest a second site of interaction between the two proteins. According to PIPE analysis it is possible that the C-terminal end of the YBL007C protein may also serve as a site of interaction.

To examine whether PIPE can detect the sites of interaction between proteins, we took 10 protein pairs (Table 3) for which their sites of interactions had previously been reported. Of the 10 protein pairs, PIPE identified 7 pairs as interactors. The sites of interactions reported by PIPE for 4 of these pairs were the same as those previously reported in the literature. It was previously shown in [30] that the region 310–768 in protein YNL243W is responsible for its interaction with amino acids 118–361 in protein YBL007C. PIPE analysis of the protein pair is shown in Figure 2. Apparent by a peak with a high score of 45, PIPE analysis indicates that the region between amino acids 350 and 410 in protein YNL243W co-occurs frequently with the region between amino acids 100 and 250 in protein YBL007C. This observation suggests that the two proteins are interacting via the mentioned regions. This is in agreement with the regions experimentally shown to mediate an interaction between YNL243W and YBL007C [30]. Interestingly, PIPE also detected a second potential site of interaction between the same region (amino acids 350–410) for YNL243W as above and the C-terminal region (amino acids 1175–1225) of YBL007C. Of interest is that previously it was shown that the C-terminal domain of YBL007C can function as a site of protein-protein interaction [31,32]. Further studies are required, however, to verify the presence of an interaction between these newly predicted sites. Furthermore, PIPE successfully determined the previously documented site of interaction between YCR084C and YBR112C. It is reported that the first 75 amino acids of YCR084C is responsible for an interaction with the N-terminal region of YBR112C [33,34]. PIPE correctly predicted an interaction between these two sites. In addition PIPE analysis successfully predicted the known interaction site between YBR079CandYNL243W[35] as well as the region responsible for dimerization of YMR159C [36].

All together, this data indicates a 40% success for PIPE to identify the previously reported interaction sites between proteins. We note that this success rate is measured from a very small data set since there is not much reliable data available that correctly identifies the sites of protein interactions.

### Ability of PIPE to detect novel protein-protein interactions

**Figure 3:**
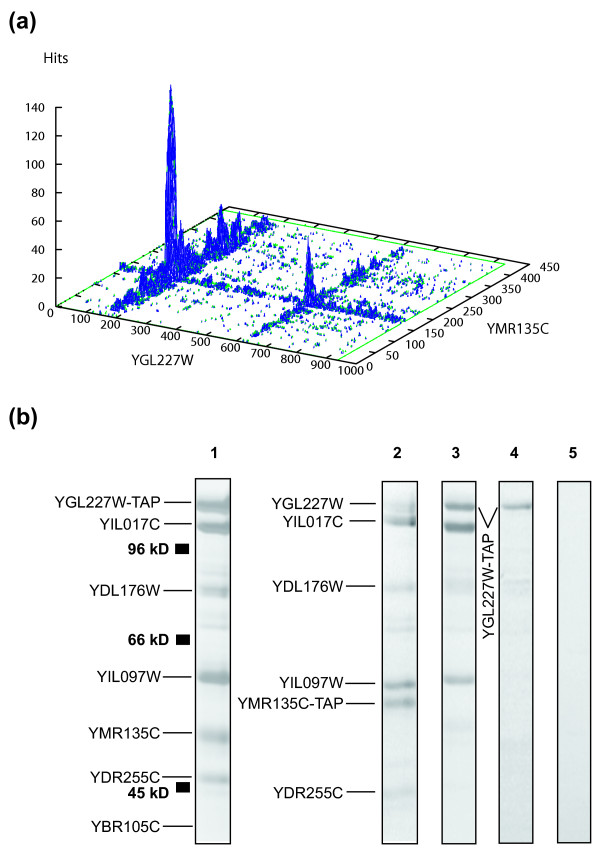
**Novel protein-protein interactions**. PIPE can successfully detect novel protein-protein interactions. With a score of 136 in (a), PIPE analysis predicts an interaction between YGL227W and YMR135C. (b) TAP-tag analysis confirms the interaction between YGL227W and YMR135C. When YGL227W is TAP-tagged, YMR135C is purified as an interacting subunit (panel 1). Reciprocal TAP-tagging of YMR135C also identifies YGL227W as an interacting partner, confirming the validity of the observed interaction between the two proteins (panel 2). Panels 3 and 4 show the purifications of TAP-tagged YGL227W strains in which either YDR255C (YDR255CΔ) or YMR135C (YMR135CΔ) were deleted, respectively. Deletion of YDR255C (panel 3) had no effect in the co-purification of other subunits. However, when YMR135C (panel 4) was deleted, the interactions between TAP-tagged YGL227W and most other subunits were eliminated. Panel 5 is used as a control and shows the purification of a strain, which is not tagged.

The ability of PIPE to detect novel protein-protein interactions was examined by analyzing the potential interaction between a novel pair of proteins, YGL227W-YMR135C for which no experimental interaction data was available when we initiated this project. Little is known about the molecular function of these genes, but the inactivation of either YGL227W or YMR135C, also known as vid30 and gid8, respectively, are shown to alter proteasome dependent catabolite degradation of fructose-1,6-bisphosphatase (FBPase) [37]. PIPE analysis of this protein pair is shown in Figure 3(a). The peak score of 140 indicates that the proteins are capable of interacting with one another. This is in agreement with the phenotypic characteristics of the yeast strains in which either YGL227W or YMR135C is deleted. Both deletion strains are incapable of degrading FBPase [37]. To confirm the validity of the observed interaction, TAP tag methodology was employed. An advantage of TAP-tagging over other generic protein-protein interaction detection assays is that it detects those interactions that occur under native level of protein expression in the cell. Therefore, TAP tag identifies those complexes that really exist *in vivo*. As shown in Figure 3(b) when YGL227W is TAP-tagged and its corresponding complex is affinity purified, YMR135C is identified as an interacting protein partner. The LC-MS MS analysis also indicated that YMR135C co-purified as an interacting partner when TAP-tagged YGL227W was purified. The reciprocal tagging and purification of YMR135C confirmed this interaction. YGL227W was identified as an interacting partner when TAP-tagged YMR135C complex was affinity purified. The presence of YGL227W in the purified mixture was also verified by LC-MS MS analysis. All together, these data demonstrate that PIPE has the ability to successfully predict novel protein-protein interactions.

### Ability of PIPE to detect novel protein-protein interactions that cannot be identified by TAP tagging

Besides the obvious advantages of PIPE over TAP tagging (speed and the ease of use), PIPE can also be used to analyse yeast proteins for which TAP tagging fails. A very recent genome-wide yeast TAP tagging project has indicated that out of the 6,466 yeast open reading frames, only 1,993 (or 31%) can be successfully TAP-tagged and purified [38]. Data from the same authors [38] suggest that TAP tagging of YCR093W was unsuccessful. However, with a score of 60, PIPE analysis successfully identified a previously known interaction between YCR093W and YPR072W [39]. Since the screening of yeast complexes to saturation using TAP tag has identified approximately 62% of the expected yeast protein complexes [38], it might be expected that a different approach like PIPE may be able to contribute to the identification of some remaining interactions.

### Ability of PIPE to elucidate the internal architecture of protein complexes

TAP tagging of YGL227W resulted in the co-purification of six other proteins (YIL017C, YMR135C, YDL176W, YIL097W, YBR105C and YDR255C) as indicated in Figure 3(b). This suggests that YGL227W forms a novel protein complex with these proteins that here we term vid30 complex (vid30c). The presence of this protein complex is further confirmed by TAP tagging of YMR135C, which resulted in the co-purification of the same constituent subunits; see Figure 3(b). The internal architecture of this protein complex, however, remains unknown, as TAP tag has a limited ability to resolve the internal structure of complexes.

**Figure 4:**
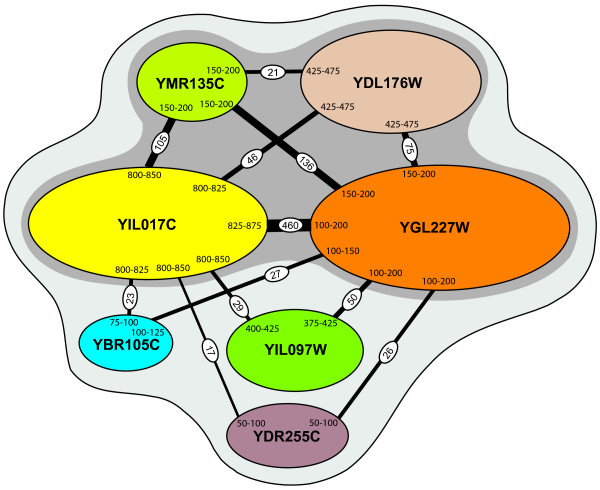
**Internal architecture of vid30c, as suggested by PIPE**. YGL227W, YIL017C, YMR135C and YDL176W all interact with each other with relatively high PIPE scores, and seem to form a core compartment of vid30c. YIL097W, YBR105C and YDR255C, with relatively lower PIPE scores, interact with YGL227W and YIL017C only, and not with each other, suggesting a secondary component of vid30c. PIPE scores are embedded within the connecting lines. The regions responsible for interactions are indicated.

To test the ability of PIPE to provide a better understanding of the internal architecture of protein complexes, we systematically analyzed protein pairs of vid30c constituent subunits using PIPE. This resulted in the analysis of 21 protein pairs, the result of which is summarized in Table 4. This data was then used to generate a hypothetical representation of how the protein subunits might be interacting. As shown in Figure 4, vid30c seem to have a core component consisting of four subunits YGL227W, YIL017C, YMR135C and YDL176W. These four subunits seem to be in direct interaction with each other. The complex also seems to have a secondary component, the members of which (YIL097W, YBR105C and YDR255C) seem to interact with YGL227W and YIL017C only and not to each other. The hypothesized interactions among the subunits of the core component seem to have high PIPE scores suggesting high affinity and likelihood for interactions. The PIPE scores associated with the secondary components, however, tend to be lower. The highest PIPE score (460) was that for the interaction between YIL017C and YIL017C, which might be expected, as all the subunits of vid30c seem to interact with these two proteins. The lowest significant PIPE score was for YDR255C, which only had two significant scores, 25 and 17, for interactions with YGL227W and YIL017C, respectively, suggesting a low affinity for an interaction with vid30c. The hypothetical sites of interactions identified by PIPE are different in size. For example, YIL017C seem to interact with a small region of YBR105C (75–100), and with a relatively broader region of YGL227W (100–200). It also seems that each protein may have a specific region responsible for interaction with protein partners. This in turn may suggest that some of these proteins may compete for an interaction with the same partner. There remains the possibility however, that the broader regions (such as YGL227W region 100–200) may support simultaneous interactions with more than one protein partners.

To experimentally examine the information from PIPE analysis about the internal topology of vid30c, we made two gene deletion strains. For this purpose YDR255C and YMR135C were selected which have similar molecular weights (50 and 52 kD, respectively). According to PIPE, YDR255C has the lowest affinity to vid30c. Therefore, it might be expected that the deletion of this gene may be insignificant to the integrity of vid30c. However, PIPE analysis placed YMR135C in the core component of vid30c. Depending on the molecular function of YMR135C, it might be expected that the elimination of this protein may (or may not) alter the formation of vid30c. Therefore, two yeast deletion strains, YDR255CΔ and YMR135CΔ, were generated in which either the YDR255C or YMR135C gene was deleted, respectively, in a TAP-tagged YGL227W yeast background. In agreement with PIPE analysis, TAP tagging of YDR255CΔ strain indicated that deletion of YDR255C showed no significant effect in the formation of vid30c; see Figure 3(b). Besides YDR255C all other members of vid30c co-purified with TAP-tagged YGL227W. However, when YMR135C was deleted (YMR135CΔ), the interactions between TAP-tagged YGL227W and most other vid30c subunits were eliminated; see Figure 3(b). This suggests that vid30c was not formed in the absence of YMR135C. This is in agreement with PIPE analysis, which indicated a low affinity between YDR255C and vid30c, but placed YMR135C in the core component of vid30c with strong affinity to this complex.

To estimate the success rate of PIPE in predicting the internal structure of protein complexes, we tested PIPE on 10 protein complexes (see Table 5). Each complex consists of three subunits, and the subunits are reported to be interacting with each other in a chain format, that is "a-b-c", where protein "a" interacts with "b" but not with "c", and protein "c" interacts with "b" only. It should be noted however, that due to the technical limitations associated with the approaches used to generate our current view of the internal structure of protein complexes and in the absence of a sufficient number of studies on the crystal structural analysis of protein complexes, the topology of the reported complexes should be considered with caution. Regardless, these 10 protein complexes generated a total of 30 potential interactions, 20 of which were shown to exist and 10 of which were shown not to. PIPE detected 13 interactions of the 20 shown to exist. It also detected 4 false/novel interactions of the 10 shown not to exist. In total, from the 10 protein complexes, PIPE detected 3 internal architectures identical to what was reported previously. It should be noted that due to the absence of more reliable data, this may not represent the true success rate of PIPE but instead represents the overlap between the existing small data set and the data generated by PIPE.

### Discussion of the algorithmic approach

As outlined in Materials and Methods, the PIPE method predicts the likelihood of interaction between two query proteins A and B by measuring how often pairs of subsequences in A and B co-occur in pairs of protein sequences in the dataset that are known to interact. The amount of computation involved is substantial. For a pair of interacting proteins, on average, several hours of computation time were required for a standard desktop machine. This time was observed to be directly proportional to the number of re-occurrences of similar sequences in different interacting proteins in our dataset of interacting protein partners. As the number of corresponding sequences that co-occurred in the dataset increased, so did the computation time associated with analyzing the target protein pair. Similarly, the computation time required for non-interacting protein pairs were observed to be significantly lower as the co-occurring sequences were absent in these pairs. For the next version of PIPE, we expect considerable speed improvements. The current version of PIPE concentrates on the predictive precision of the method and we are currently in the process of applying more sophisticated data structures and algorithms to reduce PIPE's computation time. In addition, we plan to parallelize PIPE so that it can be executed on a processor cluster instead of a single workstation, which is rather straightforward. We expect that this will provide further significant performance improvements.

## Conclusion

Here we report on the making of a computational tool, termed PIPE, which can effectively identify protein interactions among *S. cerevisiae *protein pairs. The sensitivity of this engine to identify true interactions is estimated to be 61%, which is comparable to that of the currently available generic biochemical assays used for large-scale detection of protein-protein interactions. PIPE has an estimated specificity of 89%, which is a significant improvement over the currently available confidence rates for most other assays. In addition, PIPE considerably reduces the cost associated with detecting protein interactions by traditional biochemical methods.

We are currently in the process of applying more sophisticated data structures and algorithms as well as parallel processing technology to significantly reduce PIPE's computation time. Furthermore, by incorporating additional protein interaction data into PIPE's database, as well as using more precise tools for detecting similar short polypeptide sequences in different proteins (e.g. allowing for gaps), we hope to further increase the precision of PIPE in the future. In addition, the incorporation of the data gathered from three-dimensional structures of proteins and protein complexes is also expected to further enhance the ability of PIPE to detect protein-protein interactions. The fact that protein-protein interactions can be successfully detected from the amino acid sequences of proteins alone and without additional information/predictions about the proteins can set path for the development of other such tools for predicting interactions in other organisms. We are currently in the process of modifying PIPE to predict human protein-protein interactions.

## Methods

### PIPE algorithm

Our protein-protein interaction prediction algorithm relies on previously determined interactions. At the time we initiated this study, our dataset was composed of 15118 pairs of protein-protein interactions using a total of 6304 yeast protein sequences and was compiled from the *S. cerevisiae *protein interactions reported in the DIP [40] and MIPS [41] databases. These interactions were determined using several methods, each having a limited accuracy. Since our algorithm is based on uncertain data, we expect a certain degree of error associated with our predictions.

**Figure 5:**
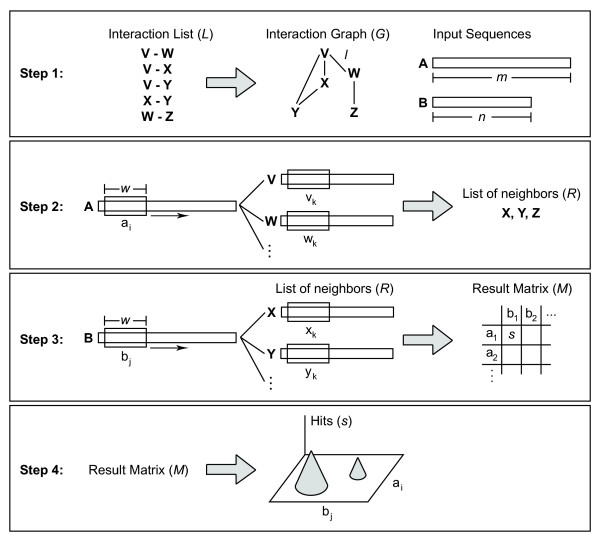
**Illustration of PIPE algorithm**. Illustration of the four main steps in the PIPE algorithm. In Step 1 we input the database as well as the input sequences A and B. Using the database we build the interaction graph G. Step 2 involves searching every fragment ai of size w in all the proteins of the database. When matches are found, the neighbours or the sequence containing the match are added to the list of neighbours R. In Step 3, we search for fragment bj in the proteins in R. If a match occurs, we increase the cell aibj in the result matrix M. Once all fragments have been processed, the result matrix M is graphed into a 3D surface, where peaks are possible sites of interaction.

The principle of our method is as follows: assume we have two query proteins A and B, along with the knowledge that certain proteins C and D are interacting. If a region (subsequence) a1 in A resembles a region in C, and a sequence b1 in B resembles a region in D, there is a possibility that A and B are also interacting via an interaction between the corresponding a1 and b1 sequences, which co-occur in both protein pairs A-B and C-D. As the number of interacting protein pairs in the database which contain the corresponding sequences a1 and b1 increases so does the likelihood that a1 and b1 are the true mediators of an interaction between A and B. The algorithm can be divided into the following steps (see also Figure 5):

**Step 1: **Input the dataset of known protein interaction (referred to as the interaction list):

**(a) **Every protein sequence in the interaction list is represented as a node in a graph G.

**(b) **Every interacting pair of sequences in the interaction list results in an edge l in G between the two respective nodes.

**(c) **Input the two query sequences: sequence A of length m and sequence B of length n.

**Step 2: **Sequence A is fragmented into overlapping segments of w amino acids each. In other words, we use a sliding window of length w and move it forward by one amino acid in each step. For each fragment a_i_, i = 1 to (m-w+1), we do the following:

**(a) **Search for fragment a_i _in every sequence in the database. We also use a sliding window of length w in every sequence in the database and for every fragment in each sequence, we use the PAM120 matrix to match the corresponding amino acids with a_i_. We define a score which is the sum of PAM120 scores for the w pairs of amino acids matched. That score will be used to identify whether two fragments are similar or not.

**(b) **For every sequence containing a fragment that matches a_i_(score equal or greater than a threshold S_pam_), we add to a list R all neighbours of that sequence in G (by following its adjacent edges in G).

**Step 3: **Once all fragments a_i _have been searched in the database and all neighbours of successful matches have been added to the list R, we search all fragments b_j _of sequence B in R. As in Step 2, we use again a sliding window of size w to create fragments b_j_, j = 1 to (n-w+1), of B and then search each bj in R. Every match of a b_j _in R will result in a score increment of one in a result matrix where each row i represents a fragment a_i _in A and each column j represents each fragment b_j _in B.

**Step 4: **The result matrix is presented as a 3D surface where the rows and columns represent the fragments a_i _and b_j_, respectively, and the elevation represents the score S, i.e. the number of matches observed for the corresponding fragments a_i _and bj.

### PIPE parameter tuning

There are three main parameters that need to be set for PIPE: (1) the window size w, (2) the threshold S_pam _that determines a match between two fragments with respect to PAM120, and (3) the threshold M for the PIPE score (number of matches observed for two fragment a_i _and b_j_) above which PIPE reports an interaction between two proteins. The three values w, S_pam _and M depend on each other. One of them can be set as a free parameter and the other two then need to be set accordingly. We chose to set the window size w to 20. Theoretically, one would want w to be as small as possible in order to identify interaction sites as precisely as possible. However, too small a window size would create too many random matches. A window size of 20 is a small value for which the probability of random matches small enough (see "Method 2" discussion below). We used two different methods to determine the values of the remaining two parameters, S_pam _and M.

Method 1: Trial and error. For a set of 20 interacting pairs and 20 non-interacting protein pairs, we tried various combinations of S_pam _and M, requiring close to 400 hours of computation time. It was observed that a PAM120 cut off score S_pam _= 35 and a threshold for the number of matches M = 10 was most selective in differentiating between interacting and non-interacting pairs.

**Figure 6:**
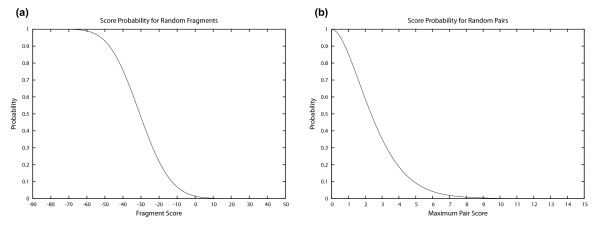
**PIPE parameter tuning**. In (a) the measured probability for two random fragments to match with a given PAM120 score (fragment score) is shown to be 10^-6 ^for scores larger than 35. This was done using 1 M random fragment pairs of length 20. In (b) 1,000 random protein pairs of length 500 are used to show that the measured probability for two random proteins to have a maximum PIPE score larger than 10 is 10^-6^.

Method 2: Statistical evaluation. To evaluate the significance of M = 10 matches observed for a PAM120 cut off score S_pam _= 35 with window size w = 20, we measured the likelihood of such an event for random sequences. First, we built 1,000,000 random fragment pairs by creating 2,000,000 random fragments of length 20 whose amino acid distribution is the same as measured for our yeast database. Figure 6(a) shows the measured probability for two random fragments to match with a given PAM120 score (fragment score). We observe that the probability for two fragments to match with a PAM120 score larger than 35 is less than 10^-6^. Next, we built 1,000 random protein pairs by creating 2,000 random proteins of length 500 whose amino acid distribution is the same as measured for our yeast database. For each protein pair, we ran PIPE and determined the maximum score in the PIPE result matrix. Figure 6(b) shows the measured probability for two random proteins to have a given maximum PIPE score. We observe that the probability of a PIPE score larger than 10 is less than 10^-6^.

### Interpretation of PIPE output

Typical graphs of non-interacting and interacting pairs are shown in Figure 1(a) and 1(b) respectively. The x and y axis represent the amino acids regions of the target proteins, starting from the N-terminal amino acid at position 1. Therefore position 5 corresponds to the 20 amino acids window starting at the fifth amino acid of the polypeptide. The score on the z axis represents the number of times that a pair of 20 amino acid sequences co-occurs in the dataset of interacting proteins. A high score corresponds to a high incidence of co-occurrence of the sequences among the database of interacting proteins. Therefore a score of 5 indicates that the corresponding sequences co-occur five times in our database, whereas a score of 50 indicates that the co-occurrence is present in 50 pairs of interacting proteins. We assume that a high score represents a soaring affinity for an interaction.

PIPE's sensitivity is calculated as (TP/(TP+FN)) [%], its specificity as (TN/(TN+FP)) [%], and its accuracy as ((TP+TN)/(TP+FN+FP+TN)) [%] where TP is the number of true positive, FN the number of false negatives, TN the number of true negatives, and FP the number of false positives. We note that a major source of false positives reported by PIPE is motifs with frequent occurrence in the database. Pairs of such motifs can have a high co-occurrence simply because they are very frequent.

### Yeast strains and purification procedure

The following yeast strains were used in this study: OshB6 MATa ura3-1 leu2-3,112 his3-11,15 trp1 ade2-1 YGL227W-TAP::TRP1 and OshB7 MATa ura3-1 leu2-3,112 his3-11,15 trp1 ade2-1 YMR135C -TAP:TRP1. TAP-tagged YGL227W and YMR135C proteins were purified as in [9,10]. In brief, the tagged proteins were affinity purified on immunoglobulin G (IgG) and calmodulin columns from extracts of yeast cells (3 liters). Half of the affinity purified complex mixture was fractioned on an SDS-PAGE and visualized (using silver staining). The protein bands were subjected to in gel trypsin digestion followed by identification using matrix associated laser desorption ionization-time-of-flight (MALDI-TOF) mass spectrometry (MS). The protein detection limitations of MALDI-TOF MS was complemented by subjecting the other half of the purified mixture to gel-free microcapillary-scale reverse-phase liquid chromatography-electrospray iontrap tandem (LC-MS) MS analysis. YDR255CΔ and YMR135CΔ yeast deletion strains were generated by one-step PCR transformation as before [10] using primer pairs: TCAGTATGAGATAAGTGTGTCTTCAAGAGAGATGCAGCACTGAGTAGGGAACCAAGAAACGCACATACGATTTAGGTGACAC, CGAGAGCAGGTTGCTAAAGGTGGTTTACTGTAGAAAACTACTGTGTTCTGTTATCGCTTCCAATAATACGACTCACTATAGGGAG and AAAGGGGCAGTAGAGACAAATATCAGCCGGATGAAGATATATTTGTGTGTGGTAACAAATAGAACACATACGATTTAGGTGACAC, CACACTCACACATGCACACGCACACACACATATATAAATATATACGTACTATGTATGAATACGACTCACTATAGGGAG, respectively.

## Availability and requirements

PIPE program was written in C++ with Linux as the operating system. The source code is subjected to the terms established by GNU and is available free of charge from the authors on request or can be downloaded from [42]. We have also set up a user-friendly WWW interface for the program at [42].

## Authors' contributions

AG, SP, FD, AC, JC contributed to the conceptual development of PIPE. SP and XL contributed to the implementation of PIPE. AD, AE, MG, JG, MJ, NK, AG contributed towards the TAP tagging experiments and data collection. All authors read and approved the final manuscript.

## Figures and Tables

**Table 1:** Positive validation set. The list of the protein pairs that our known to interact. This list was used to evaluate PIPE's accuracy to detect protein interactions.

**Protein A**	**Protein B**	**Protein A**	**Protein B**	**Protein A**	**Protein B**
YAL021C	YNR052C	YDR394W	YOR117W	YJL187C	YBR133C
YAL032C	YDR416W	YDR416W	YKL095W	YJR068W	YBR087W
YAR003W	YBR175W	YDR477W	YGL115W	YJR076C	YHR107C
YBL007C	YHR016C	YDR490C	YPL004C	YJR093C	YPR107C
YBL045C	YPR191W	YDR490C	YGR086C	YJR093C	YMR061W
YBL056W	YDR071C	YEL037C	YER162C	YKL028W	YDR311W
YBR102C	YLR166C	YER027C	YDR477W	YKL135C	YPR029C
YBR102C	YDR166C	YER027C	YGL115W	YKL135C	YPL259C
YBR103W	YIL112W	YER081W	YIL074C	YKL135C	YHL019C
YBR123C	YOR110W	YER095W	YML032C	YKL139W	YJL006C
YBR125C	YDR071C	YFL039C	YNL138W	YKR046C	YLR291C
YBR126C	YMR261C	YFR004W	YOR261C	YKR048C	YCL024W
YBR126C	YML100W	YGL112C	YBR198C	YKR068C	YBR254C
YBR135W	YDL155W	YGL190C	YML109W	YKR068C	YDR472W
YBR154C	YOR116C	YGL195W	YFR009W	YLR103C	YLR274W
YBR160W	YBR135W	YGL208W	YGL115W	YLR103C	YBR202W
YBR170C	YGR048W	YGL237C	YOR358W	YLR166C	YPR055W
YBR200W	YER114C	YGR040W	YDL159W	YLR226W	YPR161C
YBR200W	YAL041W	YGR040W	YPL049C	YLR274W	YBR202W
YBR254C	YDR472W	YGR240C	YMR205C	YLR291C	YGR083C
YCR052W	YFR037C	YGR261C	YBR288C	YLR371W	YDL203C
YCR077C	YDL160C	YHR148W	YJR002W	YLR423C	YBR217W
YCR093W	YPR072W	YHR166C	YBL084C	YLR423C	YNL182C
YDL002C	YER092W	YHR172W	YNL126W	YLR423C	YGL237C
YDL002C	YGL150C	YHR172W	YLR212C	YML077W	YDR472W
YDL092W	YKL122C	YIL021W	YOL005C	YML094W	YLR200W
YDL126C	YKL213C	YIL033C	YKL166C	YML098W	YML015C
YDL126C	YBR170C	YIL033C	YJL164C	YMR091C	YFR037C
YDL147W	YDR179C	YIL038C	YPR072W	YMR146C	YDR429C
YDL216C	YMR025W	YIL062C	YKL013C	YMR167W	YNL082W
YDR074W	YMR261C	YJL005W	YNL138W	YMR231W	YDL077C
YDR074W	YML100W	YJL008C	YOR117W	YNL126W	YLR212C
YDR148C	YFR049W	YJL124C	YBL026W	YNR006W	YHL002W
YDR179C	YMR025W				

**Table 2:** Negative validation set. The list of the protein pairs that our known not to interact. This list was used to evaluate PIPE's accuracy to detect protein interactions.

**Protein A**	**Protein B**	**Protein A**	**Protein B**	**Protein A**	**Protein B**
YAL003W	YDL232W	YCL004W	YDL168W	YLR170C	YNL137C
YAL047C	YBR243C	YCL009C	YDR077W	YLR170C	YOR346W
YAR071W	YCL030C	YCL018W	YDL078C	YLR170C	YPL048W
YBL068W	YBL099W	YCL025C	YDR079W	YLR170C	YPL147W
YBL068W	YLR170C	YCL040W	YDR086C	YLR195C	YMR203W
YBL068W	YNR006W	YCL050C	YCL057W	YLR195C	YNR006W
YBL068W	YPL029W	YCR005C	YDL022W	YLR244C	YMR203W
YBL079W	YBL090W	YCR021C	YCR053W	YLR244C	YPL147W
YBL079W	YBL099W	YCR094W	YMR170C	YMR011W	YMR203W
YBL079W	YKL122C	YDL028C	YDL066W	YMR011W	YNL137C
YBL079W	YMR203W	YDL055C	YBR243C	YMR011W	YOR025W
YBL079W	YNL137C	YDL067C	YDL212W	YMR011W	YPL048W
YBL079W	YPL147W	YDL142C	YDL159W	YMR011W	YPL147W
YBL079W	YPR133W-A	YDL236W	YDR086C	YMR203W	YNL029C
YBL084C	YBL090W	YDR001C	YDL137W	YMR203W	YNR043W
YBL084C	YBL099W	YDR044W	YCR014C	YMR203W	YOR025W
YBL084C	YLR170C	YDR144C	YAL003W	YMR203W	YPL048W
YBL084C	YNL137C	YGL055W	YLR170C	YMR203W	YPL147W
YBL090W	YGL055W	YGL055W	YNR006W	YMR261C	YNL137C
YBL090W	YKL122C	YHR041C	YDR538W	YMR261C	YNR006W
YBL090W	YLR170C	YIL021W	YBR177C	YMR261C	YOR321W
YBL090W	YMR203W	YKL122C	YKL181W	YMR261C	YPL147W
YBL090W	YOR321W	YKL122C	YLR170C	YMR261C	YPR133W-A
YBL090W	YPR133W-A	YKL122C	YLR195C	YNL029C	YNL137C
YBL099W	YGL055W	YKL122C	YMR203W	YNL029C	YNR006W
YBL099W	YKL122C	YKL122C	YNL137C	YNL029C	YOR025W
YBL099W	YLR170C	YKL122C	YOR346W	YNL029C	YPL147W
YBL099W	YNR006W	YKL122C	YPL048W	YNL137C	YNR043W
YBL099W	YOR346W	YKL122C	YPL147W	YNL137C	YOL136C
YBR208C	YCR048W	YKL181W	YLR170C	YNL137C	YOR025W
YBR218C	YDL077C	YKL181W	YMR203W	YNL137C	YPL147W
YBR236C	YCR011C	YKL181W	YNL137C	YNR006W	YOR025W
YBR237W	YDL090C	YLR170C	YMR203W	YNR006W	YPL048W
YBR248C	YDL194W				

**Table 3:** Set of interacting proteins with previously reported interaction sites. This list was used to evaluate the efficiency of PIPE to predict sites of interactions for an interacting protein pair.

**Protein A**	**Protein B**
YPL153C	YBL051C
YNL088W	YGL017W
YNL243W	YBL007C
YCR084C	YBR112C
YMR190C	YNL282W
YGL153W	YDR244W
YBR079C	YNL243W
YDR477W	YGL115W
YMR159C	YMR159C
YDR216W	YMR303C

**Table 4:** Internal PIPE scores for vid30c. PIPE scores are used to show the potential interactions between the subunits of vid30c.

**Protein A**	**Protein B**	**Score**
YBR105C	YDL176W	5
YBR105C	YDR255C	4
YBR105C	YGL227W	27
YBR105C	YIL017C	23
YBR105C	YIL097W	4
YBR105C	YMR135C	10
YDL176W	YGL227W	75
YDL176W	YIL017C	46
YDR255C	YDL176W	4
YDR255C	YGL227W	26
YDR255C	YIL017C	17
YDR255C	YIL097W	4
YDR255C	YMR135C	5
YIL017C	YGL227W	460
YIL097W	YDL176W	6
YIL097W	YGL227W	50
YIL097W	YIL017C	29
YMR135C	YDL176W	21
YMR135C	YGL227W	136
YMR135C	YIL017C	105
YMR135C	YIL097W	9

**Table 5:** Set of protein complexes with previously reported internal structures. This list was used to evaluate the efficiency of PIPE to predict the internal architecture of protein complexes. Only the adjacent subunits are reported to be interacting.

**Protein A**	**Protein B**	**Protein C**
YGR004W	YLR324W	YDL089W
YPR119W	YBR135W	YDL155W
YDR378C	YER112W	YJL124C
YMR197C	YOR036W	YBL050W
YPR185W	YGR120C	YPR105C
YKL103C	YAL034W	YGR120C
YBL026W	YDL160C	YEL015W
YLR423C	YGR113W	YIL144W
YDR084C	YGL198W	YPL095C
YLR045C	YCL029C	YER016W
